# Supplementary material for: A GNSS receiver positioning algorithm based on weighting the statistical properties of discontinuous signals at lower rotation speeds
Source: PLoS One. 2025 Dec 5;20(12):e0336707. doi: 10.1371/journal.pone.0336707 (PMC12680169; doi:10.1371/journal.pone.0336707)
Supplement: Attachment — Submitted filename: rebuttal letter.docx [file pone.0336707.s002.docx]

**Rebuttal letter**

We would like to begin with our sincere appreciation for all the valuable comments, insightful suggestions, and thoughtful corrections offered by the reviewers and editor to our manuscript ([Manuscript : A GNSS Receiver Positioning Algorithm Based on Weighting the Statistical Properties of Discontinuous Signals at Lower Rotation Speeds]). The comments and suggestions definitely helped us to improve the quality of the manuscript.

We have revised the manuscript, with major revisions marked in red in the Manuscript-Track Change. These revisions are listed below, and we have responded to the reviewers' comments point by point.

**Reviewer #1:** The minor comments are as follows:

Comments:

**1.** Introduction: ‘the PDOP value,’. Please provide the full name when the acronym is first introduced. Similar errors are present with other abbreviations in the text. Actually, the PDOP is adopted to weight the observations (see. Realistic stochastic modeling considering the PDOP and its application in real-time GNSS point positioning under challenging environments), the authors need discuss this issue before introducing the statistical properties of discontinuous in this study.

**Response:** Thanks for your advice. As per your suggestion, the PDOP value now appears with its full name the first time it is mentioned, and other similar errors have been fixed. The PDOP value is the open-root sum of the squares of the errors in latitude, longitude, elevation, etc. The factor reflecting the decay of positioning accuracy is related to the spatial geometric distribution of the measured satellites. Generally, the larger the spatial distribution range, the smaller the PDOP value and the higher the positioning accuracy. A PDOP value of less than 3 is desirable, whereas a PDOP value greater than 3 indicates lower positioning accuracy. Although PDOP values can be used for weighting observations, in this paper they are used to demonstrate the improvement in positioning accuracy achieved by the method described. The specific modifications are as follows:

During relatively low-speed rotation of the rotating carrier, the receiver can rely on capturing the finite time signal only when the antenna is turned in the direction of the satellite due to the long signal loss, a process in which the signal may be affected by noncontinuous reception. The conventional numerical preprocessing method for mitigating this is to reject the data with poor convergence as outliers. The conventional approach to this effect is to reject the poorly converged data as wild values, but for non-continuous reception environments, if it is simply rejected, however, this will reduce the available satellite data and also affect the satellite geometric distribution, which further deteriorates the positioning results. In this paper, we propose a Global Navigation Satellite System (GNSS) receiver positioning algorithm based on weighting the statistical characteristics of discontinuous signals at lower rotational speeds. The method references the preorder available positioning results and employs a Kalman filtering algorithm designed based on independent model identification of observation weights to fully utilize the observation data of each satellite and improve the positioning accuracy. According to experimental data, the horizontal positioning error is reduced from 37.16 m with the original positioning algorithm to 28.31 m with the proposed method at 1/18 r/s, as an example.

The traditional method in this non-continuous reception environment, which itself has less available satellite observation data, simply culls the data, which can easily cause the number of available satellite observation data to further decrease, plus this sudden decrease in the number of satellites can also affect the satellite geometric distribution, i.e., the Position Dilution of Precision (PDOP) value, causing further deterioration in the positioning results. PDOP is the value of the open root sign of the sum of the squares of the errors in latitude, longitude and elevation.

**2.** Introduction: The research topic of this paper belongs to the stochastic modeling in GNSS community. Recently, the stochastic modeling of positioning under complex environments is also a hot topic, such as ‘A composite stochastic model considering the terrain topography for real-time GNSS monitoring in canyon environments’. This issue also needs to be clarified.

**Response:** Thanks for your advice. The issue of A composite stochastic model considering the terrain topography for real-time GNSS monitoring in canyon environments has been clarified. The focus of this paper is missile flight, rocket lift-off and similar high-altitude operations, where the terrain has less impact. The specific modifications are as follows:

The positioning of rotating carriers remains a key area of research in satellite navigation technology. Nowadays, the positioning of rotating carriers remains a focus of research in satellite navigation technology. For example, rockets, artillery shells and missiles flying in the air rarely have the problem of satellite signal blocking. However, when these carriers spin to control their attitude during flight, the reception of satellite navigation signals becomes discontinuous, which affects the continuity and accuracy of their positioning. This phenomenon is more obvious when the carrier's rotational speed is low.

The main solution to this type of problem is currently still to use positioning algorithms for satellite navigation to optimize under single-antenna conditions. In the traditional single-antenna solution, although the accurate continuous attitude estimation algorithm based on a single antenna can improve the attitude accuracy, the improved accuracy is limited. Under a certain rotational speed, the receiver can rely on its own baseband loop to maintain the signal tracking state and complete the localization solution, which is similar to a conventional receiver. However, at relatively low rotational speeds, the receiver can rely only on the limited time signal when the antenna is turned to the satellite direction to complete the signal tracking and convergence, which may be impacted by short signal reception times, i.e., noncontinuous reception, which may not be enough time to complete the capture. Furthermore, the signal may be lost again after capture when it is too late for the convergence of the observation data. For this issue, the conventional numerical preprocessing method can be used to address the effects of poorly converged data by eliminating them as outliers. The traditional method in this non-continuous reception environment, which itself has less available satellite observation data, simply culls the data, which can easily cause the number of available satellite observation data to further decrease, plus this sudden decrease in the number of satellites can also affect the satellite geometric distribution, i.e., the Position Dilution of Precision (PDOP) value, causing further deterioration in the positioning results. PDOP is the value of the open root sign of the sum of the squares of the errors in latitude, longitude and elevation.

**3.** 3.3 Using the mean square ⋯ is calculated: ‘where K_k is the filter gain.’. Please provide the specific formula for K_k.

**Response:** Thanks for your advice. The specific modifications are as follows:

| $K_{k}=P_{k}^{-}C^{T}(CP_{k}^{-}C^{T}+R)^{-1}$ | | (12) |
| --- | --- | --- |
|  |  | (13) |

where $K_{k}$ is the filter gain, $R$ is the covariance matrix of the measurement noise vector $v_{k}$.

**4.** Equation (18): The inequality ‘i≥2’ should be placed to the right of the formula ‘T_i=2τ_k T_(i-1) (τ_k )-T_(i-2) (τ_k )’ as it is part of this equation.

**Response:** Thanks for your advice. The specific modifications are as follows:

Then, the Chebyshev polynomial $T_{i}\left(\tau_{k}\right)$ is:

| $\begin{matrix} T_{0}(\tau_{k})=1 \\ T_{1}(\tau_{k})=\tau\\ T_{i}=2\tau_{k}T_{i-1}\left(\tau_{k}\right)-T_{i-2}\left(\tau_{k}\right) \end{matrix}\begin{matrix} \\ \\ i\geq2 \end{matrix}$ | (19) |
| --- | --- |

**5.** Figure 5: The legend placement obstructs the main content of the graph, and the placement is inconsistent. Consider placing a single legend for the three subplots to address this issue.

**Response:** Thanks for your advice. The legend has been modified to one to prevent blocking of the figure. The specific modifications are as follows:

**Fig 5**. Comparison of elevation localization results of this algorithm at varying rotational speeds. Schematic diagram. (a) Comparison of elevation positioning results at 1/4 r/s. (b) Comparison of the elevation positioning results at 1/12 r/s. (c) Comparison of the elevation positioning results at 1/18 r/s.

**6.** Figure 6: In other figures, the original and proposed methods are represented in red and blue, respectively. However, in this figure, the colors are uniform, which might confuse the readers. Consider aligning the colors of these two methods with those used in other figures for consistency.

**Response:** Thanks for your advice. The specific modifications are as follows:

|   (a) |   (b) |
| --- | --- |
|   (c) |   (d) |
|   (e) |   (f) |

**Reviewer #2:** I have several concerns:

**1.** The introduction is not well-written. The limitations of previous work are not described and not clearly highlighted. Additionally, the contributions of the proposed approach are not made clear.

**Response:** Thank you for your advice. The comment definitely helped us to improve the quality of the manuscript. The limitations of the previous work and the contribution of the proposed methodology have been added according to the comment. The specific changes are as follows:

The positioning of rotating carriers remains a key area of research in satellite navigation technology. Nowadays, the positioning of rotating carriers remains a focus of research in satellite navigation technology. For example, rockets, artillery shells and missiles flying in the air rarely have the problem of satellite signal blocking. However, when these carriers spin to control their attitude during flight, the reception of satellite navigation signals becomes discontinuous, which affects the continuity and accuracy of their positioning. This phenomenon is more obvious when the carrier's rotational speed is low.

The main solution to this type of problem is currently still to use positioning algorithms for satellite navigation to optimize under single-antenna conditions. In the traditional single-antenna solution, although the accurate continuous attitude estimation algorithm based on a single antenna can improve the attitude accuracy, the improved accuracy is limited. Under a certain rotational speed, the receiver can rely on its own baseband loop to maintain the signal tracking state and complete the localization solution, which is similar to a conventional receiver. However, at relatively low rotational speeds, the receiver can rely only on the limited time signal when the antenna is turned to the satellite direction to complete the signal tracking and convergence, which may be impacted by short signal reception times, i.e., noncontinuous reception, which may not be enough time to complete the capture. Furthermore, the signal may be lost again after capture when it is too late for the convergence of the observation data. For this issue, the conventional numerical preprocessing method can be used to address the effects of poorly converged data by eliminating them as outliers. The traditional method in this non-continuous reception environment, which itself has less available satellite observation data, simply culls the data, which can easily cause the number of available satellite observation data to further decrease, plus this sudden decrease in the number of satellites can also affect the satellite geometric distribution, i.e., the Position Dilution of Precision (PDOP) value, causing further deterioration in the positioning results. PDOP is the value of the open root sign of the sum of the squares of the errors in latitude, longitude and elevation.

In this study, the low rotational speed environment is examined, and related research directions are referenced. The commonly used patch antenna has an actual signal coverage angle of approximately 160°. By mounting two symmetrically placed antennas, the satellite signals can be captured in all directions. This approach has the potential to reduce the number of antennas needed to address the challenges of previous approaches while maintaining sufficient signal coverage in low-speed environments. In this non-continuous reception environment, where there are inherently fewer available satellite observations, the use of the weighted approach described in this paper not only improves the problem of positioning accuracy, but also improves the satellite geometric distribution. According to experimental data, the horizontal positioning error is reduced from 37.16 m with the original positioning algorithm to 28.31 m with the proposed method at 1/18 r/s, as an example. Refer to the Experimental verification section for the improvement of the satellite geometric distribution.

**2.** The paper lacks a clear organization. The structure of the paper is not outlined, leaving readers without guidance on how the content is arranged.

**Response:** Thank you for your advice. The comment definitely helped us to improve the quality of the manuscript. The specific changes are as follows:

This thesis is a GNSS receiver positioning algorithm based on the statistical characteristics of discontinuous signals at weighted lower rotation speeds, so this thesis first describes the signal characteristics of low rotation speed GNSS signals in the case of a single antenna, and then weights the standard Kalman filtering algorithm according to the signal characteristics, so that the influence of abnormal satellite signals on the positioning results will be reduced, i.e. the weight will be reduced, so as to achieve improvements in the positioning results and satellite geometric distribution. Finally, the method of this paper is experimentally verified, and the improvement of positioning results is reflected by the horizontal error and elevation error, and the improvement of satellite geometric distribution is reflected by the number of satellite receipts and PDOP value.

**3.** The literature study is insufficient and not defined properly. It fails to provide a comprehensive comparison with relevant existing methods or to position the proposed work within the current state of research.

**Response:** Thank you very much for your suggestions. They will certainly help us to improve the quality of our manuscripts. In response to the third suggestion, the detailed explanation is as follows:

Currently, the main solution to this type of problem remains optimization under single antenna conditions. For example, using satellite navigation positioning algorithms and upgrading the antenna to improve signal capture and make the signal more continuous. In this paper, we mainly use patch antennas, which are commonly used in rotating carrier, and the accuracy of a single patch antenna combined with a positioning algorithm to solve this type of problem is limited. In this paper, we use a dual antenna combined with a weighted Kalman filtering positioning algorithm to achieve the improvement of positioning accuracy and geometric distribution of satellites. Therefore, this paper is more concerned with describing the antennas and explaining in detail that the use of dual antennas is the most appropriate in the research environment of this paper.

**4.** The description of the proposed technique is incomplete. Section 3 focuses on the basics of the Kalman Filter, which are standard and do not contribute novel insights. Several basic equations are unnecessarily included; these should be removed to focus more on derivations relevant to the proposed method.

**Response:** Thank you very much for your suggestions. They will certainly help us to improve the quality of our manuscripts. The detailed explanation is as follows:

The introduction to Section 3 of this paper is a practical application of the Kalman filter in our experimental scenario, which includes real-world situations that are not identical to the basic principles of the Kalman filter. Second, in Section 3.4 of Section 3, it is the core method of this paper, the weight setting, which implements the weighted Kalman localization algorithm through the weight setting.

**5.** Equation numbers are provided but are not referenced or used effectively within the text, making their inclusion seem redundant.

**Response:** Thank you very much for your suggestions. They will certainly help us to improve the quality of our manuscripts. The detailed explanation is as follows:

The third part of the paper, as far as possible, is written in the order of the formulas, with the latter formula being a continuation of the previous one and therefore less cited. However, lines 278 and 283 inevitably cite the previous formula.

**6.** The heading "Experimental Verification" is misplaced. The entire experimental procedure should be described before verification, allowing readers to understand the context of the experiments.

**Response:** Thank you very much for your suggestions. They will certainly help us to improve the quality of our manuscripts. The detailed explanation is as follows:

This paper's experimental procedure uses the dual-antenna test environment shown in Fig. 4 to set the rotary table speed and collect relevant original data. It then uses the software shown in Fig. 5 for subsequent algorithm verification.

To verify the viability of the proposed localization method, a rotating carrier test-bed with dual antennas and an application environment are established, as illustrated in Fig 4.

|  |  |
| --- | --- |
| (a) | (b) |
|  |  |
| (c) | (d) |

Fig 4. Schematic diagram of the experimental scenario. (a) Rotating carrier flat view. (b) Rotating carrier top view. (c) Antenna. (d) Self-developed chip navigation receiver board.

Based on the experimental scenario diagram illustrated in Fig 4, the dual antenna positioning technique is employed for signal reception. Dual antennas are mounted on the side of the rotating carrier, and the antennas are symmetrically mounted on both sides, enabling at least one antenna to receive satellite signals during rotation. This expands the range of the received signals and makes capturing satellite signals easier.

The above experimental scenarios were used to collect the low rotational speed rotation data of the rotating carrier, and the data was played back using the self-developed software shown in Fig. 5 to achieve the validation of the methodology of this paper.

Fig 5. Experimental verification software interface diagram.

**7.** The dataset for figures from Figure 5 onwards is not provided, and there is no explanation on how the epochs were executed. This lack of detail is also evident in Figure 7, where crucial information is missing.

**Response:** Thank you very much for your suggestions. They will certainly help us to improve the quality of our manuscripts. The detailed explanation is as follows:

A epoch refers to the point in time at which data is updated in the satellite signal or the time period during which the receiver acquires the data. I apologize for the inconvenience. The data I provided earlier was in its raw form. You will now need parsed data for each chart. I have added the parsed data. For Figure 7, the horizontal coordinate represents the epoch, while the vertical coordinate represents the dilution of precision value position.

**8.** The results are presented in a very general manner, without detailed explanations or in-depth interpretation. This section requires significant elaboration to be meaningful.

**Response:** Thank you very much for your suggestions. They will certainly help us to improve the quality of our manuscripts. The detailed explanation is as follows:

In this research paper, the weighted positioning algorithm based on Chebyshev polynomials is deeply explored and analyzed. The law of satellite navigation received signal during low-speed rotation combined with the influence of the installation method and number of rotating carrier antennas on the satellite navigation positioning performance is analyzed, and it is proposed that under the premise of satisfying the receiving star coverage capability, the use of as few antennas as possible should be given priority, i.e. two antennas is the optimal solution. In addition, a Kalman filter algorithm based on independent model identification of observation weights is proposed. The algorithm uses Chebyshev extrapolation algorithm to judge the range of data accuracy, formulate reasonable weight function rules, and classify data with different accuracy. The receiver state equation based on the GNSS positioning system is established, the receiver state is predicted based on the state transfer matrix, and the mean square error array of the a priori estimation, the residual of the observation and the measurement variance are calculated. The Chebyshev polynomials are used to achieve the solution of the extrapolated pseudorange value, and the Chebyshev coefficient matrix is determined by the least-squares method to provide the weight setting for the positioning solution. According to the extrapolated pseudo-distance value, the equivalent weight function is set, and reasonable weights are assigned to the data of different states, and then the new a posteriori estimation error of the mean square error array and measurement variance are obtained, so as to realize the accurate measurement update of the state estimation value. Then the test platform is designed, with the help of the dual-antenna rotating carrier test platform and application environment, and the dual-antenna positioning technology is adopted to ensure that at least one of the antennas can receive satellite signals in the rotating process, to expand the range of the received signals, and to improve the success rate of satellite signal capture. The traditional filtering algorithm and the weighted positioning algorithm with Chebyshev polynomials are comprehensively tested, including the test of elevation positioning accuracy, the test of horizontal error, the test of the number of available satellites and the test of PDOP value. By comparing the experimental results of the two algorithms under different rotational speeds, the weighted positioning algorithm with Chebyshev polynomials visually demonstrates the significant advantages of the Chebyshev polynomials in terms of positioning accuracy, stability of error, utilization of the number of available satellites, and optimization of the PDOP value, and thus fully verifies the feasibility and effectiveness of the algorithm.

Although the algorithm in this paper has improved the positioning accuracy under the condition of low rotational speed, the actual application scenarios are complicated and diverse, such as urban high-rise buildings, mountainous terrain and other environments, the satellite signals are easily blocked and reflected, resulting in the multipath effect, and the algorithm does not fully take into account the impact of these complex environmental factors on the positioning accuracy, and the positioning performance under such environments needs to be further researched and verified. In-depth study of the propagation characteristics of satellite signals in complex environments (e.g., urban canyons, mountainous areas, indoor areas, etc.) is needed to establish a more accurate signal model, incorporate the multipath effect and signal obstruction into the algorithm, and further optimize the weighted positioning algorithm based on Chebyshev polynomials, so as to improve the accuracy and reliability of the algorithm, and to enable it to be better applied to practical scenarios, such as urban navigation and emergency rescue. The algorithm is based on Chebyshev polynomials.

**9.** The novelty of the proposed work is a major concern. The manuscript does not present a clear, innovative approach, and the method does not appear to offer substantial improvements over existing techniques.

**Response:** Thank you very much for your suggestions. They will certainly help us to improve the quality of our manuscripts. The detailed explanation is as follows:

1)The environment of the application

Current research focuses more on scenarios where rotating carriers are in highly dynamic environments, and less has been done to improve localization in low speed scenarios. However, low speed positioning accuracy also needs improvement.

2.	Improvement of solutions

The capture and stable tracking of satellite signals in various scenarios has been a controversial issue. There are two solutions to this problem for today's scientists, the first is to use a higher quality antenna and the second is to increase the power of the receiver, which undoubtedly increases the cost.

In this paper, two antennas are used instead, and then a modified algorithm is used to improve the problem of low localization accuracy in low-speed environments. No additional cost and effective improvement.

3.	Improvements in localization algorithms

1)	In this paper, Chebyshev polynomials are used to process observed data from satellite signals.

2)	In this paper, Chebyshev polynomials are combined with weight functions to further process the satellite observation data.

3)	Based on the processing results of the second step, the equivalent weights are obtained and then combined with the Kalman filtering algorithm, the algorithm is weighted to achieve the final result of improved localization accuracy.
