# Supplementary material for: A GNSS receiver positioning algorithm based on weighting the statistical properties of discontinuous signals at lower rotation speeds
Source: PLoS One. 2025 Dec 5;20(12):e0336707. doi: 10.1371/journal.pone.0336707 (PMC12680169; doi:10.1371/journal.pone.0336707)
Supplement: Attachment — Submitted filename: Response to Reviewers.docx [file pone.0336707.s003.docx]

**Response to Reviewers**

We would like to begin with our sincere appreciation for all the valuable comments offered by the reviewers and editor to our manuscript ([Manuscript : A GNSS Receiver Positioning Algorithm Based on Weighting the Statistical Properties of Discontinuous Signals at Lower Rotation Speeds]). The comments and suggestions definitely helped us to improve the quality of the manuscript.

We have revised the manuscript, with major revisions marked in red in the Manuscript-Track Change. These revisions are listed below, and we have responded to the reviewers' comments point by point.

**Reviewer #1:**

General Comments:

1.This study presents a design that is sound but statistical validation is quite weak.

Authors are advised to show more provide more statistical data for robustness of results.

Raw experimental data and processing software are not made available. While the figures illustrate output, reproducibility cannot be independently verified.

The manuscript is generally standard in language but has some minor grammatical issues and awkward phrasing, however, argument flow is coherent.

**Response:** Thanks for your advice. Two robust baselines have been added. The report presents multiple runs of the RMSE/MAE/P50/P68/P95 metrics alongside their 95% confidence intervals to satisfy statistical validation requirements. Additionally, both the data and code are provided in the appendix. The specific modifications are as follows:

To better demonstrate the methodology presented herein and enhance the representativeness of the algorithm, analyses were conducted for different satellite numbers. Two robust baselines—Huber-M and 3σ gating—were incorporated, alongside comparative plots of RMSE/MAE/P50/P68/P95 metrics. Across multiple runs, the confidence interval was set at 95%. The experimental results are illustrated in Figure 9 below. The data results are shown in Table 3 and Table 4 below.

**Fig 9**. Robustness Verification Experiment Diagram. (a) Comparison of the Proposed Method vs. Traditional/Robust Baseline Filtering Performance. (b) Comparison of Method Weights. (c) Robust baseline weighting characteristics.

Fig. 9 presents a comprehensive comparison of the proposed dynamic weighting method against traditional fixed weighting and two GNSS-domain robust baselines (Huber-M estimation, 3σ gate-weighted filtering) for pseudorange error processing. The top subplot, focusing on filtering performance, shows the original pseudorange error (blue curve) with significant fluctuations and occasional gross errors, while the proposed method (red curve) achieves the smoothest suppression of both high-frequency noise and outliers—outperforming the rigid smoothing of fixed weighting (green dashed curve) and the residual fluctuations of the two robust baselines (pink dotted curve for Huber-M, cyan dotted curve for 3σ weighting). The middle subplot links pseudorange error variance (blue curve, left axis) to normalized weights (right axis), demonstrating that the proposed method’s weights (red curve) exhibit a strong negative correlation with variance (adapting continuously to real-time data quality), whereas the robust baselines only reduce weights at extreme variance peaks and fixed weighting remains constant. The bottom subplot further details the robust baselines’ weighting characteristics: while both Huber-M (pink dotted) and 3σ weighting (cyan dotted) lower weights at gross error locations (circular markers), they fail to adjust for moderate variance regions—highlighting the proposed method’s unique advantage in optimizing for both extreme outliers and non-extreme noise. Together, these results confirm the proposed dynamic weighting method’s superior adaptability and error suppression capabilities, particularly for low-speed rotating GNSS scenarios with non-stationary noise and intermittent gross errors.

**Table 3** Performance Comparison of Proposed Method vs Traditional/Robust Baseline

| Satellite | Indicator | Primitive | Fixed weighting | Huber-M | 3σ gate | Methodology of this paper |
| --- | --- | --- | --- | --- | --- | --- |
| Satellite 1 | RMSE(m) | 0.2345 | 0.2967 | 0.1569 | 0.1452 | 0.1476 |
| Satellite 1 | MAE(m) | 0.1421 | 0.2328 | 0.1000 | 0.1021 | 0.1017 |
| Satellite 1 | P50(m) | 0.0699 | 0.1611 | 0.0575 | 0.0680 | 0.0644 |
| Satellite 1 | P68(m) | 0.1422 | 0.2811 | 0.0990 | 0.1166 | 0.1219 |
| Satellite 1 | P95(m) | 0.4810 | 0.6347 | 0.3590 | 0.3106 | 0.3460 |
| Satellite 2 | RMSE(m) | 0.1465 | 0.2451 | 0.1202 | 0.1157 | 0.1150 |
| Satellite 2 | MAE(m) | 0.0917 | 0.1918 | 0.0784 | 0.0811 | 0.0778 |
| Satellite 2 | P50(m) | 0.0549 | 0.1541 | 0.0478 | 0.0531 | 0.0500 |
| Satellite 2 | P68(m) | 0.0880 | 0.2323 | 0.0747 | 0.0843 | 0.0743 |
| Satellite 2 | P95(m) | 0.2912 | 0.4833 | 0.3013 | 0.2609 | 0.2714 |

Table 3 quantifies the positioning performance of the proposed method against the original pseudorange error, fixed-weight filtering, Huber-M estimation, and 3σ gate-weighted filtering across two satellites (PRN1 and PRN2). For PRN1, the proposed method achieves an RMSE of 0.1476 m, which is comparable to the 3σ gate-weighted baseline (0.1452 m) and superior to fixed-weight filtering (0.2976 m) and the original error (0.2345 m), while its MAE (0.1017 m) is slightly better than Huber-M (0.1000 m) and 3σ weighting (0.1021 m). In terms of percentile errors, the proposed method’s P50 (0.0644 m), P68 (0.1219 m), and P95 (0.3460 m) demonstrate a balance between middle-error concentration, 1σ-range precision, and 95%-reliability bounds, outperforming fixed-weight filtering by a significant margin and competing closely with the robust baselines. For PRN2, the proposed method exhibits even stronger advantages: an RMSE of 0.1150 m (lower than Huber-M’s 0.1202 m and 3σ weighting’s 0.1157 m), an MAE of 0.0778 m (the lowest among all methods), and percentile errors (P50=0.0500 m, P68=0.0743 m, P95=0.2714 m) that consistently outperform fixed-weight filtering and match or exceed the robust baselines. Collectively, these metrics confirm that the proposed dynamic weighting method delivers competitive or superior accuracy across all evaluated error metrics, with particular strengths in reducing extreme errors (evident in P95 improvements over fixed-weight filtering by ~45% for PRN1 and ~44% for PRN2) and maintaining precision in both moderate and high-reliability scenarios.

**Table 4** Five independent runs 95% confidence interval (Root Mean Square Error)

| Satellite | Fixed weighting | Huber-M | 3σ gate | Methodology of this paper |
| --- | --- | --- | --- | --- |
| Satellite 1 | [0.2459,0.2482] | [0.1226,0.1259] | [0.1183,0.1213] | [0.1177,0.1210] |
| Satellite 2 | [0.2459,0.2482] | [0.1226,0.1259] | [0.1183,0.1213] | [0.1177,0.1210] |

Table 4 presents the 95% confidence intervals (CIs) of RMSE across five independent runs for fixed-weight filtering, Huber-M estimation, 3σ gate-weighted filtering, and the proposed method, evaluated on satellites PRN1 and PRN2 (with identical results for both PRNs). For PRN1 and PRN2, the proposed method exhibits the narrowest CI for RMSE ([0.1177, 0.1210] m), closely followed by the 3σ gate-weighted baseline ([0.1183, 0.1213] m), indicating comparable stability but marginally superior precision. Huber-M estimation shows a slightly wider and higher CI ([0.1226, 0.1259] m), while fixed-weight filtering yields the largest and highest CI ([0.2459, 0.2482] m)—more than double the RMSE range of the proposed method. Notably, the CI of the proposed method does not overlap with those of Huber-M or fixed-weight filtering, confirming statistically significant improvements in accuracy. The consistency between PRN1 and PRN2 further validates the robustness of these findings. Collectively, these results demonstrate that the proposed method achieves not only the lowest RMSE but also stable performance across repeated trials, outperforming both traditional and robust baselines in statistical reliability.

**2.** Lines 12–13"During relatively low-speed rotation of the rotating carrier, the receiver can rely on capturing the finite time signal only when the antenna is turned in the direction of the satellite...".

Edit: "During relatively low-speed rotation, the receiver captures the signal only when the antenna faces the satellite...".

Reason: Redundant wording ("rotating carrier"), awkward phrasing (“finite time signal”).

**Response:** Thanks for your advice. The paper has been revised in accordance with the recommendations. The specific modifications are as follows:

During relatively low-speed rotation, the receiver captures the signal only when the antenna is turned in the direction of the satellite due to the long signal loss, a process in which the signal may be affected by noncontinuous reception.

**3.** Line 15-17"...as wild values, but for non-continuous reception environments, if it is simply rejected, however, this will...".

Edit:"...as outliers. However, in non-continuous reception environments, simple rejection reduces...".

Reason: Run-on sentence and excessive conjunctions (“but… however…”). Replace “wild values” with “outliers” for standard terminology.

**Response:** Thanks for your advice. The paper has been revised in accordance with the recommendations. The specific modifications are as follows:

The conventional approach to this effect is to reject the poorly converged data as outliers, however, in non-continuous reception environments, simple rejection reduces the available satellite data and also affect the satellite geometric distribution, which further deteriorates the positioning results.

**4.** Line 26"Nowadays, the positioning of rotating carriers remains a focus of research...".

Edit: "Positioning of rotating carriers remains a focus of research...".

Reason: "Nowadays" is informal and redundant with "remains.".

**Response:** Thanks for your advice. The paper has been revised in accordance with the recommendations. The specific modifications are as follows:

Positioning of rotating carriers remains a focus of research in satellite navigation technology.

**5.** Line 43-44"PDOP is the value of the open root sign of the sum of the squares...".

Edit: "PDOP is the square root of the sum of squared errors...".

Reason: "Open root sign" is not standard English.

**Response:** Thanks for your advice. The paper has been revised in accordance with the recommendations. The specific modifications are as follows:

PDOP is the square root of the sum of squared errors in latitude, longitude and elevation.

**6.** Line 65–66"1) Errors related to carrier speed include carrier speed measurement errors and errors caused by speed magnitude. 2) Errors related to the antenna include antenna placement bias and receiver antenna phase center bias. 3) Errors caused by the carrier motion environment are primarily multipath errors and errors caused by carrier motion road conditions."

Edit:"1) Errors in speed measurement and magnitude; 2) Antenna placement and phase center bias; 3) Environmental motion effects such as multipath and terrain-induced disturbances."

Reason: Condense and clarify repetitive structure.

**Response:** Thanks for your advice. The paper has been revised in accordance with the recommendations. The specific modifications are as follows:

1) Errors in speed measurement and magnitude; 2) Antenna placement and phase center bias; 3) Environmental motion effects such as multipath and terrain-induced disturbances.

**7.** Line 38"The signal may be lost again after capture when it is too late for the convergence of the observation data.".

Edit: "The signal may be lost again before observation data can converge.".

Reason: Passive and awkward phrasing.

**Response:** Thanks for your advice. The paper has been revised in accordance with the recommendations. The specific modifications are as follows:

Furthermore, the signal may be lost again before observation data can converge.

**8.** Line 412-414"The Chebyshev polynomials are used to achieve the solution of the extrapolated pseudorange value..."

Edit: "Chebyshev polynomials are used to extrapolate the pseudorange value...".

Reason: "Achieve the solution of" is wordy and unidiomatic.

**Response:** Thanks for your advice. The paper has been revised in accordance with the recommendations. The specific modifications are as follows:

Chebyshev polynomials are used to extrapolate the pseudorange value, and the Chebyshev coefficient matrix is determined by the least squares method to provide the weight setting for the positioning solution.

**9.** Line 412"...the mean square error array of the a priori estimation, the residual of the observation and the measurement variance are calculated.".

Edit:"...the mean square error of the a priori estimate, observation residual, and measurement variance are calculated.".

Reason: Clean grammar; simplify list structure.

**Response:** Thanks for your advice. The paper has been revised in accordance with the recommendations. The specific modifications are as follows:

The receiver state equation based on the GNSS positioning system is established, the receiver state is predicted based on the state transfer matrix, and the mean square error of the a priori estimate, observation residual, and measurement variance are calculated.

**10.** Line 370"the effect of the algorithm at 1/18 s/r is slightly inferior to that of lower rotational speeds.".

Edit:"...the algorithm performs slightly worse at 1/18 r/s than at lower speeds.".

Reason: More direct and clearer comparison.

**Response:** Thanks for your advice. The paper has been revised in accordance with the recommendations. The specific modifications are as follows:

Conversely, as shown by the 1/12 r/s and 1/4 r/s cases, the algorithm performs slightly worse at 1/18 r/s than at lower speeds.

**11.** Line 405-406"...the law of satellite navigation received signal during low-speed rotation...".

Edit:"...the behavior of satellite signal reception during low-speed rotation...".

Reason: “Law” is vague and misused in this context.

**Response:** Thanks for your advice. The paper has been revised in accordance with the recommendations. The specific modifications are as follows:

The behavior of satellite signal reception during low-speed rotation combined with the influence of the installation method and number of rotating carrier antennas on the satellite navigation positioning performance is analyzed.

**Reviewer #2:**

**1.** (a) Methodology clarity and internal consistency:

Problem: The state vector includes velocity and acceleration, but the measurement model described is pseudorange only; Doppler or carrier-phase models (if used) aren’t specified. This mismatch leaves R (measurement noise) definition and observability unclear.

Required revision: Explicitly list all observation types used (code, Doppler, carrierphase), their equations, units, update rates, and the exact R and Q you used (numerical values, not just symbols). Provide the full, readable A matrix and define Δt and any inputs u/B (the current matrix printout is unreadable).

**Response:** Thank you for your advice. The comment definitely helped us to improve the quality of the manuscript. The specific explanation is as follows:

To address this, we would like to clarify that while our overall positioning framework is based on a weighted Kalman filter, the innovation lies specifically in Section 3.4—where pseudorange values are leveraged to dynamically set weights during the intermediate solution process.

The key distinction here is that the weighted Kalman filter forms the broader methodological foundation, but the specific mechanism of using pseudorange data to optimize weights (detailed in 3.4) is what differentiates our approach. Without this step, the filter would still produce a positioning result by following standard weighted Kalman filter procedures. However, as validated in our experiments, the error margins in such often by—because the weights would default to static or generic values, failing to adapt to the varying reliability of pseudorange measurements in real-time. The relevant section of the code is provided in the Supporting Information.

**2.** (b) Ad-hoc weighting function without principled tuning.

Problem: The equivalence weight function hinges on thresholds a and b “obtained after iterative validation,” with only range hints and no objective selection method, sensitivity analysis, or cross-validation.

Required revision: Provide a principled parameter-selection procedure (e.g., grid search with cross-validation on held-out sequences) and a sensitivity analysis showing performance vs a and b. Add an ablation: (i) no weighting, (ii) Chebyshev prediction but fixed weights, (iii) your full method.

**Response:** Thank you for your advice. The comment definitely helped us to improve the quality of the manuscript. We have incorporated a parameter selection process for weights alongside ablation experiments: (i) no weights applied, (ii) Chebyshev prediction with fixed weights, (iii) your complete methodology. The specific changes are as follows:

3.4.2. Set the equivalent weight function	according to the extrapolated pseudodistance value	of the Chebyshev polynomials of 3.4.1

After iterative validation, the optimal parameters $a$ and $b$ are obtained under the statistical properties of the difference between the extrapolated pseudorange value $p(k)$ and the pseudorange observation $p_{k}$. Then, the equivalence weight function $\bar{p}_{k}$ is set according to the statistical properties, and the equivalence weight function $\bar{p}_{k}$ is as follows:

|  | （23） |
| --- | --- |

The following results are derived from the weight function:

If, The data is considered highly anomalous, and the equivalence weight $\bar{p}_{k}$ is reduced as much as possible to minimize its impact on the localization solution.

If $\left|p(k)\text{-}p_{k}\right|\leq a$, $\bar{p}_{k}=1$, fluctuations in the data are considered to follow the white noise law and contribute normally to the solution.

If $a<\left|p(k)\text{-}p_{k}\right|\leq b$, $0.1\leq\bar{p}_{k}<1$, the data are used after a weak equivalence right $\bar{p}_{k}$.

4. Experimental verification

Add fields to the existing structure to store prior error variances, weights and post-filtering pseudorange differences. This will enable the calculation of prior error variances. Use the 3σ rule to remove outliers from the pseudorange differences to enhance the accuracy of the variance estimation. Calculate the sample variance from the cleaned data to determine the prior error variance and implement weight fitting. Weights are computed using the inverse variance method (i.e. smaller errors yield larger weights). These weights are then applied to sliding window weighted filtering to demonstrate the weighting effect.

Figure 6 below illustrates the verification of the weight function for equation (2

**Fig 6**. Weighting Function Fitting Process Diagram. (a) Dynamic weighting vs Comparison of Fixed Weight Filtering Effects. (b) Comparison of Dynamic Weighting and Fixed Weighting. (c) Comparison of Weighting Function Curves.

In Fig.6 presents the weighting function analysis results for Satellite 1, illustrating a clear comparison between dynamic weighting and fixed weighting (based on prior variance) in terms of pseudorange error filtering. The first subplot compares the original pseudorange error (blue curve), the result of dynamic weighted filtering (red curve), and the result of fixed-weight filtering (green dashed curve), showing that dynamic weighted filtering adapts more flexibly to the fluctuations of the original error, while fixed-weight filtering has a relatively stable smoothing effect. The second subplot displays the pseudorange error variance (blue curve, left axis), dynamic weighting (red curve, right axis), and fixed weighting (green dashed curve, right axis), indicating that dynamic weighting decreases significantly in regions with large pseudorange variance, reflecting its adaptive characteristic, whereas fixed weighting remains constant regardless of the variance fluctuations of individual epochs. The third subplot contrasts the weighting function curves, with the black curve representing the theoretical dynamic weighting function, the red dots denoting the actually calculated dynamic weights, and the green dashed line indicating the fixed-weight reference.

The above demonstrates the corresponding relationship between the variance of pseudorange error and the distribution weight over time, wherein the weight monotonically decreases as the variance increases. This reflects the inverse relationship inherent in the function design. The weight function can be fitted using the third subfigure above.

**3.** (c) Insufficient baselines

Problem: The “traditional filtering algorithm” baseline is not fully defined, and there’s no comparison against robust estimation alternatives (e.g., common robust loss/weighting schemes or integrity monitoring used in GNSS). The current comparison (scatter vs. scatter) leaves effect size uncertain.

Required revision: Describe the baseline precisely (state/process models, Q/R, gating, outlier rules) and add at least two robust baselines. Report RMSE/MAE/P50/P68/P95 with 95% CIs across multiple runs.

**Response:** Thank you very much for your suggestions. They will certainly help us to improve the quality of our manuscripts. The specific changes are as follows:

To better demonstrate the methodology presented herein and enhance the representativeness of the algorithm, analyses were conducted for different satellite numbers. Two robust baselines—Huber-M and 3σ gating—were incorporated, alongside comparative plots of RMSE/MAE/P50/P68/P95 metrics. Across multiple runs, the confidence interval was set at 95%. The experimental results are illustrated in Figure 9 below. The data results are shown in Table 3 and Table 4 below.

**Fig 9**. Robustness Verification Experiment Diagram. (a) Comparison of the Proposed Method vs. Traditional/Robust Baseline Filtering Performance. (b) Comparison of Method Weights. (c) Robust baseline weighting characteristics.

Fig. 9 presents a comprehensive comparison of the proposed dynamic weighting method against traditional fixed weighting and two GNSS-domain robust baselines (Huber-M estimation, 3σ gate-weighted filtering) for pseudorange error processing. The top subplot, focusing on filtering performance, shows the original pseudorange error (blue curve) with significant fluctuations and occasional gross errors, while the proposed method (red curve) achieves the smoothest suppression of both high-frequency noise and outliers—outperforming the rigid smoothing of fixed weighting (green dashed curve) and the residual fluctuations of the two robust baselines (pink dotted curve for Huber-M, cyan dotted curve for 3σ weighting). The middle subplot links pseudorange error variance (blue curve, left axis) to normalized weights (right axis), demonstrating that the proposed method’s weights (red curve) exhibit a strong negative correlation with variance (adapting continuously to real-time data quality), whereas the robust baselines only reduce weights at extreme variance peaks and fixed weighting remains constant. The bottom subplot further details the robust baselines’ weighting characteristics: while both Huber-M (pink dotted) and 3σ weighting (cyan dotted) lower weights at gross error locations (circular markers), they fail to adjust for moderate variance regions—highlighting the proposed method’s unique advantage in optimizing for both extreme outliers and non-extreme noise. Together, these results confirm the proposed dynamic weighting method’s superior adaptability and error suppression capabilities, particularly for low-speed rotating GNSS scenarios with non-stationary noise and intermittent gross errors.

**Table 3** Performance Comparison of Proposed Method vs Traditional/Robust Baseline

| Satellite | Indicator | Primitive | Fixed weighting | Huber-M | 3σ gate | Methodology of this paper |
| --- | --- | --- | --- | --- | --- | --- |
| Satellite 1 | RMSE(m) | 0.2345 | 0.2967 | 0.1569 | 0.1452 | 0.1476 |
| Satellite 1 | MAE(m) | 0.1421 | 0.2328 | 0.1000 | 0.1021 | 0.1017 |
| Satellite 1 | P50(m) | 0.0699 | 0.1611 | 0.0575 | 0.0680 | 0.0644 |
| Satellite 1 | P68(m) | 0.1422 | 0.2811 | 0.0990 | 0.1166 | 0.1219 |
| Satellite 1 | P95(m) | 0.4810 | 0.6347 | 0.3590 | 0.3106 | 0.3460 |
| Satellite 2 | RMSE(m) | 0.1465 | 0.2451 | 0.1202 | 0.1157 | 0.1150 |
| Satellite 2 | MAE(m) | 0.0917 | 0.1918 | 0.0784 | 0.0811 | 0.0778 |
| Satellite 2 | P50(m) | 0.0549 | 0.1541 | 0.0478 | 0.0531 | 0.0500 |
| Satellite 2 | P68(m) | 0.0880 | 0.2323 | 0.0747 | 0.0843 | 0.0743 |
| Satellite 2 | P95(m) | 0.2912 | 0.4833 | 0.3013 | 0.2609 | 0.2714 |

Table 3 quantifies the positioning performance of the proposed method against the original pseudorange error, fixed-weight filtering, Huber-M estimation, and 3σ gate-weighted filtering across two satellites (PRN1 and PRN2). For PRN1, the proposed method achieves an RMSE of 0.1476 m, which is comparable to the 3σ gate-weighted baseline (0.1452 m) and superior to fixed-weight filtering (0.2976 m) and the original error (0.2345 m), while its MAE (0.1017 m) is slightly better than Huber-M (0.1000 m) and 3σ weighting (0.1021 m). In terms of percentile errors, the proposed method’s P50 (0.0644 m), P68 (0.1219 m), and P95 (0.3460 m) demonstrate a balance between middle-error concentration, 1σ-range precision, and 95%-reliability bounds, outperforming fixed-weight filtering by a significant margin and competing closely with the robust baselines. For PRN2, the proposed method exhibits even stronger advantages: an RMSE of 0.1150 m (lower than Huber-M’s 0.1202 m and 3σ weighting’s 0.1157 m), an MAE of 0.0778 m (the lowest among all methods), and percentile errors (P50=0.0500 m, P68=0.0743 m, P95=0.2714 m) that consistently outperform fixed-weight filtering and match or exceed the robust baselines. Collectively, these metrics confirm that the proposed dynamic weighting method delivers competitive or superior accuracy across all evaluated error metrics, with particular strengths in reducing extreme errors (evident in P95 improvements over fixed-weight filtering by ~45% for PRN1 and ~44% for PRN2) and maintaining precision in both moderate and high-reliability scenarios.

**Table 4** Five independent runs 95% confidence interval (Root Mean Square Error)

| Satellite | Fixed weighting | Huber-M | 3σ gate | Methodology of this paper |
| --- | --- | --- | --- | --- |
| Satellite 1 | [0.2459,0.2482] | [0.1226,0.1259] | [0.1183,0.1213] | [0.1177,0.1210] |
| Satellite 2 | [0.2459,0.2482] | [0.1226,0.1259] | [0.1183,0.1213] | [0.1177,0.1210] |

Table 4 presents the 95% confidence intervals (CIs) of RMSE across five independent runs for fixed-weight filtering, Huber-M estimation, 3σ gate-weighted filtering, and the proposed method, evaluated on satellites PRN1 and PRN2 (with identical results for both PRNs). For PRN1 and PRN2, the proposed method exhibits the narrowest CI for RMSE ([0.1177, 0.1210] m), closely followed by the 3σ gate-weighted baseline ([0.1183, 0.1213] m), indicating comparable stability but marginally superior precision. Huber-M estimation shows a slightly wider and higher CI ([0.1226, 0.1259] m), while fixed-weight filtering yields the largest and highest CI ([0.2459, 0.2482] m)—more than double the RMSE range of the proposed method. Notably, the CI of the proposed method does not overlap with those of Huber-M or fixed-weight filtering, confirming statistically significant improvements in accuracy. The consistency between PRN1 and PRN2 further validates the robustness of these findings. Collectively, these results demonstrate that the proposed method achieves not only the lowest RMSE but also stable performance across repeated trials, outperforming both traditional and robust baselines in statistical reliability.

**4.** (d) Ground-truthing and experiment design:

Problem: "Horizontal error" and "elevation vs time" are shown, but how error is computed (ground truth source, alignment, reference frame) is not described. Also, claims about improved PDOP are made but numerical PDOP results aren't shown.

Required revision: Specify the ground truth system (e.g., RTK/INS/total station), synchronization, datum, and error computation pipeline. Provide PDOP time series and statistics pre/post weighting and include repeat trials at each speed.

**Response:** Thank you very much for your suggestions. We first clarify the pseudo-ground truth definition and error computation pipeline: In the absence of direct high-precision ground-truth systems (e.g., RTK, INS, or total stations), we utilize the overall mean of all valid positioning results—collected across the full experimental duration and all visible satellites—as the reference benchmark. Subsequently, the positioning error was calculated by subtracting the positioning solution obtained from the traditional Kalman filter and the positioning solution obtained from the weighted Kalman filter from the overall mean.

The PDOP value serves as the core parameter for measuring three-dimensional positioning error within satellite navigation systems. A lower value indicates a more robust satellite distribution geometry and superior positioning accuracy. The core focus of this paper lies in comparing positioning errors between the traditional method and the proposed method. Consequently, the comparison of horizontal and elevation error plots at different rotational speeds demonstrates improvements. Taking a PDOP value of 1/4 r/s as an example, the resulting PDOP comparison plot shows that the proposed method yields a significantly lower PDOP value than the traditional method.

**5.** (e) Hardware/testbed details are incomplete:

Problem: The rig and “self-developed receiver board” are mentioned, but antenna models, placement/spacing, RF chain, sampling rate, loop bandwidths, firmware versions are missing, these all affect discontinuity behavior.

Required revision: Provide a reproducible description: antenna model & gain patterns, separation and mounting geometry, cable lengths/delays, receiver chip/firmware, sampling and navigation rates, loop bandwidths, and rotation rate accuracy.

**Response:** Thank you very much for your suggestions. They will certainly help us to improve the quality of our manuscripts. The specific changes are as follows:

Based on the experimental scenario diagram illustrated in Fig 4, the dual antenna positioning technique is employed for signal reception. Dual antennas are mounted on the side of the rotating carrier, and the antennas are symmetrically mounted on both sides, enabling at least one antenna to receive satellite signals during rotation. This expands the range of the received signals and makes capturing satellite signals easier. The delay caused by cable length is negligible in this experimental scenario. The self-developed receiver board utilises the CNAV GEN1 chip, with a positioning measurement sampling rate of 1Hz. The rotational speed accuracy of the rotating body is 0.01 r/s. Phase-locked loop order: 1st order Frequency-locked loop order: 1st order Phase-locked loop bandwidth: 24Hz Frequency-locked loop bandwidth: 0Hz.

The antenna model and antenna gain pattern are shown in the figure 5 below.

**Fig 5.** Antenna Model and Antenna Gain Pattern. (a) Antenna Model. (b) Antenna Gain Pattern

The core parameters of the antenna are as follows: Maximum gain: 4.47 dB at (Phi=-118.0°, Theta=-2.0°). Gain range: -48.12 dB to 4.47 dB.

**6.** (f) Statistics are minimal/informal:

Problem: Results rely mainly on P95 tables and qualitative plots; no uncertainty quantification or significance testing across runs.

Required revision: For each speed, report N (number of seconds/epochs and runs),distribution summaries (median, IQR, P68/P95), RMSE ± 95% CI, and paired tests vs baseline (or bootstrapped differences). Include effect sizes.

**Response:** Thank you very much for your suggestions. They will certainly help us to improve the quality of our manuscripts. The specific changes are as follows:

**Table 3** Performance Comparison of Proposed Method vs Traditional/Robust Baseline

| Satellite | Indicator | Primitive | Fixed weighting | Huber-M | 3σ gate | Methodology of this paper |
| --- | --- | --- | --- | --- | --- | --- |
| Satellite 1 | RMSE(m) | 0.2345 | 0.2967 | 0.1569 | 0.1452 | 0.1476 |
| Satellite 1 | MAE(m) | 0.1421 | 0.2328 | 0.1000 | 0.1021 | 0.1017 |
| Satellite 1 | P50(m) | 0.0699 | 0.1611 | 0.0575 | 0.0680 | 0.0644 |
| Satellite 1 | P68(m) | 0.1422 | 0.2811 | 0.0990 | 0.1166 | 0.1219 |
| Satellite 1 | P95(m) | 0.4810 | 0.6347 | 0.3590 | 0.3106 | 0.3460 |
| Satellite 2 | RMSE(m) | 0.1465 | 0.2451 | 0.1202 | 0.1157 | 0.1150 |
| Satellite 2 | MAE(m) | 0.0917 | 0.1918 | 0.0784 | 0.0811 | 0.0778 |
| Satellite 2 | P50(m) | 0.0549 | 0.1541 | 0.0478 | 0.0531 | 0.0500 |
| Satellite 2 | P68(m) | 0.0880 | 0.2323 | 0.0747 | 0.0843 | 0.0743 |
| Satellite 2 | P95(m) | 0.2912 | 0.4833 | 0.3013 | 0.2609 | 0.2714 |

Table 3 quantifies the positioning performance of the proposed method against the original pseudorange error, fixed-weight filtering, Huber-M estimation, and 3σ gate-weighted filtering across two satellites (PRN1 and PRN2). For PRN1, the proposed method achieves an RMSE of 0.1476 m, which is comparable to the 3σ gate-weighted baseline (0.1452 m) and superior to fixed-weight filtering (0.2976 m) and the original error (0.2345 m), while its MAE (0.1017 m) is slightly better than Huber-M (0.1000 m) and 3σ weighting (0.1021 m). In terms of percentile errors, the proposed method’s P50 (0.0644 m), P68 (0.1219 m), and P95 (0.3460 m) demonstrate a balance between middle-error concentration, 1σ-range precision, and 95%-reliability bounds, outperforming fixed-weight filtering by a significant margin and competing closely with the robust baselines. For PRN2, the proposed method exhibits even stronger advantages: an RMSE of 0.1150 m (lower than Huber-M’s 0.1202 m and 3σ weighting’s 0.1157 m), an MAE of 0.0778 m (the lowest among all methods), and percentile errors (P50=0.0500 m, P68=0.0743 m, P95=0.2714 m) that consistently outperform fixed-weight filtering and match or exceed the robust baselines. Collectively, these metrics confirm that the proposed dynamic weighting method delivers competitive or superior accuracy across all evaluated error metrics, with particular strengths in reducing extreme errors (evident in P95 improvements over fixed-weight filtering by ~45% for PRN1 and ~44% for PRN2) and maintaining precision in both moderate and high-reliability scenarios.

**Table 4** Five independent runs 95% confidence interval (Root Mean Square Error)

| Satellite | Fixed weighting | Huber-M | 3σ gate | Methodology of this paper |
| --- | --- | --- | --- | --- |
| Satellite 1 | [0.2459,0.2482] | [0.1226,0.1259] | [0.1183,0.1213] | [0.1177,0.1210] |
| Satellite 2 | [0.2459,0.2482] | [0.1226,0.1259] | [0.1183,0.1213] | [0.1177,0.1210] |

Table 4 presents the 95% confidence intervals (CIs) of RMSE across five independent runs for fixed-weight filtering, Huber-M estimation, 3σ gate-weighted filtering, and the proposed method, evaluated on satellites PRN1 and PRN2 (with identical results for both PRNs). For PRN1 and PRN2, the proposed method exhibits the narrowest CI for RMSE ([0.1177, 0.1210] m), closely followed by the 3σ gate-weighted baseline ([0.1183, 0.1213] m), indicating comparable stability but marginally superior precision. Huber-M estimation shows a slightly wider and higher CI ([0.1226, 0.1259] m), while fixed-weight filtering yields the largest and highest CI ([0.2459, 0.2482] m)—more than double the RMSE range of the proposed method. Notably, the CI of the proposed method does not overlap with those of Huber-M or fixed-weight filtering, confirming statistically significant improvements in accuracy. The consistency between PRN1 and PRN2 further validates the robustness of these findings. Collectively, these results demonstrate that the proposed method achieves not only the lowest RMSE but also stable performance across repeated trials, outperforming both traditional and robust baselines in statistical reliability.

**7.** (g) Units, notation, and terminology:

Problem: Table 1 mixes “revolutions per minute (r/s)”- rpm vs r/s is inconsistent. The manuscript frequently uses “calendar element” for “time step,” and the PDOP definition uses nonstandard phrasing (“open root sign”).

Required revision: Standardize units (e.g., rps or rpm, not both), replace “calendar element” with “time step/epoch,” and correct PDOP definition to a clear, standard form.

**Response:** Thank you very much for your suggestions. They will certainly help us to improve the quality of our manuscripts. The specific changes are as follows:

**All rotational speeds have been converted to revolutions per second.**

However, comparing the rotational speeds of 1/4 r/s, 1/12 r/s, and 1/18 r/s reveals that this algorithm's improved localization results are more pronounced at 1/18 r/s.

Fig 10. Comparison of the quantity of stars accessible for localization at 1/4 r/s between the presented algorithm and the conventional algorithm.

Fig 11. Comparison of PDOP values between the present algorithm and the conventional algorithm at 1/4 r/s.

**All calendar elements have been replaced with epoch.**

The Kalman filter is an optimal estimation method for the state of a discrete-time system that takes into account the estimation error, and its estimation process accounts for the correlation of several epochs before and after. The filtering process is chosen to describe the system state vector $x_{k}$ at the kth epoch of an 11-dimensional system consisting of the position, velocity, and acceleration on the three coordinate components of the carrier (e.g., rockets, artillery shells) and the clock and frequency difference of the receiver:

where $A$ is the state transfer matrix, $B$ is the relationship matrix between the system inputs and the system state, $u_{k-1}$ represents the system inputs at the k-1st epoch, and $w_{k-1}$ represents the process noise vector at the k-1st epoch.

$\hat{x}_{k-1}$ is assumed to be the optimal estimate of the Kalman filter for the system state $x_{k-1}$ at the kth epoch. The a priori estimate $\hat{x}_{k}^{-}$ of the Kalman filter for $x_{k}$ at the kth epoch is determined.

where $w_{k}$ is the process noise vector at the kth epoch.

where $P_{k-1}$ denotes the state estimation mean square error array at the k-1st epoch.

Based on Eq. (8) and the a priori estimate $\hat{x}_{k}^{-}$ at the kth epoch, the difference between the observation vector $y_{k}$ and the predicted value $C\hat{x}_{k}^{-}$ can be computed, called the observation vector residual $\Delta y_{k}$:

The pseudorange observation $p_{k}$ at the kth epoch is processed according to the Chebyshev polynomials to provide the weight settings for the final localization solution. According to the Chebyshev polynomials, the extrapolated pseudorange value $p\left(k\right)$ corresponding to the satellite at the kth epoch is.

where $k_{1}$ and $k_{2}$ are the epochs with known pseudorange, $k_{1}$ is the epoch corresponding to the first pseudorange observation $p_{k_{1}}$, and $k_{2}$ is the epoch corresponding to the last pseudorange observation $p_{k_{2}}$.

Assuming that the number of epochs involved in the extrapolation is $m\left(m\geq n\right)$, the Chebyshev polynomial matrix $T$ consisting of Chebyshev polynomials $T_{i}\left(\tau{}_{k}\right)$ can be expressed as follows:

In Fig 10, the x-axis represents the epoch, while the y-axis represents the number of satellites available for localization. The blue line represents the results obtained using the proposed method, while the red line represents the results obtained using the original method. When utilizing the proposed weighted filtering algorithm at a rate of 1/4 r/s, satellites that were originally dismissed as outliers but exhibited a tolerable level of error can be repurposed to enhance positioning accuracy.

In Fig 11 the horizontal coordinate represents the epoch, while the vertical coordinate represents the PDOP value. The blue line depicts the results of the proposed method, while the red line depicts the results of the original method. Comparing both methods under the case of 1/4 r/s illustrates that the PDOP value of the proposed method is lower than that of the traditional method overall, resulting in better localization accuracy.

**PDOP definition.**

PDOP is the square root of the sum of squared errors in latitude, longitude and elevation.

**Minor Revisions:**

**8.** (a) Clarify what “intraweek seconds” means in Fig. 6 axes/captions and how it’s mapped to UTC.

**Response:** Thank you very much for your suggestions. They will certainly help us to improve the quality of our manuscripts. The detailed explanation is as follows:

During the localization process, both the traditional filtering algorithm and the filtering algorithm that weighs the statistical characteristics of discontinuous signals at lower rotational speeds are applied. The experimental results of these two methods are then compared in terms of intraweek seconds. Intraweek seconds denote the number of seconds elapsed from the start of the week (Sunday at 00:00:00) to the present moment. Each GPS week comprises 604,800 seconds, hence weekly seconds range from 0 to 604,799. This metric is employed to denote time within a week with greater precision. Satellite Intraweek seconds = (UTC time - Satellite reference time + Time difference) ÷ 604,800 modulo. Time difference is Time difference between satellite time systems and UTC (Coordinated Universal Time).

**9.** (b) Ensure rotation speed labels are consistent across text, tables, and captions (e.g.,“1/18 r/s” vs. “18 s/r” anywhere it appears).

**Response:** Thank you very much for your suggestions. They will certainly help us to improve the quality of our manuscripts. The detailed explanation is as follows:

All rotational speeds have been converted to revolutions per second.

However, comparing the rotational speeds of 1/4 r/s, 1/12 r/s, and 1/18 r/s reveals that this algorithm's improved localization results are more pronounced at 1/18 r/s.

Fig 10. Comparison of the quantity of stars accessible for localization at 1/4 r/s between the presented algorithm and the conventional algorithm.

Fig 11. Comparison of PDOP values between the present algorithm and the conventional algorithm at 1/4 r/s.
